# Acute kidney injury during pregnancy leads to increased sFlt-1 and sEng and decreased renal T regulatory cells in pregnant rats with HELLP syndrome

**DOI:** 10.1186/s13293-020-00331-6

**Published:** 2020-09-24

**Authors:** Jamie Szczepanski, Shauna-Kay Spencer, Ashley Griffin, Teylor Bowles, Jan Michael Williams, Patrick B. Kyle, John Polk Dumas, Sarah Araji, Kedra Wallace

**Affiliations:** 1 Department of Obstetrics & Gynecology, University of Mississippi Medical Center, 2500 North State St, Jackson, MS 39216 USA; 2 Program in Neuroscience, University of Mississippi Medical Center, Jackson, MS USA; 3 Department of Pharmacology & Toxicology, University of Mississippi Medical Center, Jackson, MS USA; 4 Department of Pathology, University of Mississippi Medical Center, Jackson, MS USA

## Abstract

**Background:**

The incidence of acute kidney injury (AKI) during pregnancy precedes a high maternal mortality rate of 20–40%. AKI during pregnancy has multiple etiologies; however, the more common are maternal hypertensive disorders, which include preeclampsia and HELLP (hemolysis, elevated liver enzyme, low platelet) syndrome. Therefore, we sought to assess the impact of AKI on blood pressure, kidney injury, and anti-angiogenic factors during pregnancies with and without HELLP syndrome.

**Methods:**

On gestational day (GD) 12, mini-osmotic pumps were inserted into a subset of normal pregnant (NP) rats infusing 4.7 μg/kg soluble fms-like tyrosine kinase-1 (sFlt-1) and 7 μg/kg soluble endoglin (sEng) to induce HELLP syndrome. On GD18, the renal pedicles were occluded for 45 min to induce AKI via bilateral ischemia reperfusion in a subset of NP (*n* = 18) or HELLP (*n* = 20) rats. Control NP (*n* = 20) and HELLP (*n* = 20) rats underwent a SHAM surgery on GD18. Plasma, urine, and maternal organs were saved for further analysis. Renal injury was assessed via renal histopathology, glomerular filtration rate (GFR), T cell infiltration, and assessment of kidney injury molecule-1 (KIM-1) and neutrophil gelatinase-associated lipocalin (NGAL). Data was measured via two-way analysis of variance with Tukey’s test for post hoc analysis.

**Results:**

Blood pressures were increased in HELLP+AKI rats (*p* = 0.0001); both NP+AKI and HELLP+AKI rats had increased lactate dehydrogenase (*p* < 0.0001) and aspartate aminotransferase levels (*p* < 0.0001), and decreased platelet levels (*p* < 0.001) vs. NP rats. HELLP+AKI (*p* = 0.002) and HELLP rats (*p* = 0.0002) had evidence of renal fibrosis vs. NP rats. GFR was decreased in HELLP+AKI (*p* = 0.01) rats vs. NP rats. Urinary KIM-1 was increased in NP+AKI rats vs. NP (*p* = 0.003) and HELLP rats (*p* = 0.01). HELLP+AKI rats had increased urinary KIM-1 vs. NP (*p* = 0.0008) and HELLP rats (*p* = 0.004) and increased NGAL vs. HELLP rats (*p* = 0.002). HELLP+AKI rats had increased sFlt-1 (*p* = 0.009) vs. NP rats. NP+AKI (*p* = 0.02) and HELLP+AKI (*p* = 0.007) rats had increased sEng vs. NP rats. CD3^+^CD4^+^ T cells were significantly increased in HELLP+AKI rats vs. NP (*p* = 0.0002) and NP+AKI (*p* = 0.05) rats. T regulatory cells were significantly decreased in HELLP+AKI (*p* = 0.03) and NP+AKI (*p* = 0.02) rats vs. NP rats; there were no changes between groups in T helper 17 cells (*p* = 0.34).

**Conclusion:**

The findings in this study suggest that AKI during pregnancy contributes to increased blood pressure and biochemical markers for HELLP syndrome, creates an anti-angiogenic imbalance, and exacerbates kidney injury as shown on histopathology, GFR, and kidney injury markers.

## Introduction

Disorders of pregnancy such as preeclampsia, HELLP (hemolysis, elevated liver enzyme, low platelet) syndrome, and thrombotic microangiopathies are among the primary contributors to the development of acute kidney injury (AKI) during pregnancy [1, 2]. Despite the once declining rates of AKI during pregnancy (PR-AKI), the incidence of PR-AKI or the early postpartum period has recently seen a resurgence with rates ranging as high as 20% depending on the geographic location of the high-risk pregnancy [3, 4]. Increased maternal age at the time of pregnancy, and increased provider awareness and detection of renal injury and existence of multiple maternal comorbidities such as diabetes have all been proposed to contribute to the increase in PR-AKI [5, 6]. While the causes for the development of PR-AKI can be widespread, hypertensive pregnancies remain a leading cause for renal injury [5].

HELLP syndrome is considered to be a rare but potentially deadly complication of pregnancy, affecting 10–20% of women with preeclampsia [7, 8]. HELLP syndrome when complicated with AKI increases maternal mortality and morbidity compared to patients affected by HELLP alone [9, 10]. Previous studies have reported up to 60% of women with HELLP develop AKI and that while most women with pregnancy-related AKI will recover normal renal function in the postpartum period, some continue to have evidence of persistent renal injury [11, 12]. Despite these alarming facts, the lack of proper animal models limits the mechanistic studies that can be conducted to understand the underlying mechanisms that lead to the development of AKI in HELLP syndrome or contribute to maternal and fetal morbidities as well as lead to the progression of AKI to chronic kidney disease.

Soluble fms-like tyrosine kinase-1 (sFlt-1) and soluble endoglin (sEng) are anti-angiogenic proteins associated with preeclampsia and HELLP syndrome whose levels have been found to directly correlate with disease severity [13, 14]. Furthermore, studies by our lab and others have reported that infusion or overexpression of sFlt-1 and sEng leads to the HELLP syndrome phenotype in pregnant rats [13, 15, 16]. Interestingly, sFlt-1 and sEng expression has been found to be increased in patients with chronic renal diseases and in experimental rodents following ischemia reperfusion (IR) [17–19]. The purpose of the current study was to use experimental animal models to assess the effects of AKI on blood pressure, kidney injury, and anti-angiogenic factors in both normal pregnant rats and those with HELLP syndrome.

## Methods

All studies were performed in timed-pregnant Sprague-Dawley female rats (Charles River, Frederick, MD). Animals were housed in a temperature-controlled room with a 12:12 reverse light:dark cycle. All experimental procedures used in this study were in accordance with the National Institutes of Health guidelines for use and care of animals and approved by the Institutional Animal Care and Use Committee (IACUC) at the University of Mississippi Medical Center.

### Induction of HELLP and AKI

On gestational day (GD) 12, mini-osmotic pumps (model 2002, Alzet Scientific Corporation, Cupertino, CA) were inserted into a subset of normal pregnant rats (NP) to allow infusion of sFlt-1 and sEng (4.7 and 7 μg/kg, respectively, R&D Systems, Minneapolis, MN) as previously described to induce the HELLP syndrome phenotype [15, 16, 20]. In the current study, we induced AKI by IR, since it has been reported to consistently induce AKI in experimental pregnant and non-pregnant animal models [21, 22]. To induce AKI, on GD18, a subset of NP (*n* = 18) and HELLP rats (*n* = 20) were anesthetized with isoflurane and placed on an AIMS™ Thermo-controlled surgical platform prior to an abdominal midline incision being made to expose the kidneys. IR was performed via isolation and occlusion of the renal pedicles with microvascular clamps for 45 min, followed by clamp removal and closure of the abdomen with suture [23]. A lack of pulsation in the renal pedicles and the fading of kidneys from bright red to a dark purple/black color confirmed adequate ischemia. NP (*n* = 20) and HELLP (*n* = 20) rats not undergoing AKI received SHAM abdominal surgeries at this time. All rats received carotid catheters for mean arterial pressure (MAP) measurement at this same time. Once animals had recovered from anesthesia, they were placed in metabolic cages with water and standard rat chow (0.4% sodium) for overnight urine collection. The following day, the total volume of urine was recorded and urine was stored at − 20 °C.

On GD19, pressure transducers (Cobe III Transducer CDX Sema, Birmingham, AL) were connected to the carotid catheters and MAP was monitored and recorded continuously for 30 min as previously described [15]. Following MAP assessment, whole blood was collected to determine lactate dehydrogenase (LDH), aspartate aminotransferase (AST), and platelet counts. Whole blood was also collected for enzyme-linked immunosorbent assay (ELISA) experiments and to assess circulating levels of blood urea nitrogen (BUN; Vet AXCEL Chemistry Analyzer) and creatinine (CellBioLabs, San Diego, CA). Maternal organs (kidney, liver, spleen, placenta, and brains) and pups were weighed and stored at − 80 °C for further analysis or discarded.

#### Renal histopathology was used to determine the degree of renal fibrosis as AKI is associated with renal fibrosis

Kidneys (*n* = 6/group) collected at GD19 were fixed in 10% buffered formalin for 48 h and then stored in 70% ethanol at room temperature until processed for paraffin embedding. Once embedded, tissues were sectioned at 4 μm, mounted on glass slides, and stained with Masson’s trichrome to assess the degree of renal fibrosis. Ten images around the cortex were captured and scored. The degree of renal fibrosis (percentage of the image stained blue) was assessed using the NIS-Elements D 3.0 software after images were captured using a Niko Eclipse 55i microscope (Nikon, Melville, NY) [24].

#### Glomerular filtration rate was measured to determine if IR decreased renal function

On GD18, while under anesthesia for the AKI or SHAM procedure, a subset of rats (*n* = 4/group) also had jugular catheters inserted. The following day, these rats were anesthetized with isoflurane and hair from the upper back was removed via an electronic blade and depilation cream (~ 3 min) before a glomerular filtration rate(GFR) probe (NIC-Kidney, Mannheim Pharma & Diagnostics, Mannheim, Germany) was attached to the rat’s back with an adhesive sticker and secured with a backpack. An extension catheter was inserted into the jugular catheter, and the rat was allowed to recover from anesthesia. Once the rat was awake and mobile, the probe recorded a standard filtration baseline rate for 15 min followed by a 5 mg/100 g BW bolus infusion of FITC-sinistrin (Frensenius Kabi Austria INC I&D) via the jugular catheter. Continuous fluorescence was measured for 2 h, and the clearance curves were calculated using MPD Lab Ver 1.0RC3 software and the one-compartment half-life (*t*_1/2_) model where *t*_1/2_ is converted to GFR (mL/min/100 g BW) [25].

#### Urinary renal injury markers

Kidney injury molecule-1 (KIM-1; Boster Biological Technology) and neutrophil gelatinase-associated lipocalin (NGAL) were measured via ELISA based on the manufacturer’s instructions in urine samples (*n* = 10/group). KIM-1 and NGAL are proteins that have been reported to be increased in response to early kidney injury or following ischemia [26, 27]. KIM-1 and NGAL were corrected with urinary creatinine levels so that data is expressed as protein of interest (pg/mg creatinine) (BioAssay Systems, Hayward, CA). Albuminuria was measured via a commercially available kit following the manufacturer’s instructions (Sigma Aldrich, St. Louis, MO; *n* = 8/group).

#### Circulating markers and immune cell kidney infiltration were measured to determine if there was a systemic and local immune response

Commercially available enzyme-linked immunosorbent assays (R&D Systems; Millipore, Temecula, CA) were used to measure plasma levels (*n* = 7–8/group) of sFlt-1 and sEng. Plasma creatinine was measured with the creatinine kit from BioAssay Systems.

Fresh kidneys (*n* = 5/group) were minced with a razor blade, followed by digestion with 0.125 U DNaseI (Sigma) and 1 mg/mL collagenase IV (Invitrogen) for 45 min at 37 °C. After centrifugation, the single cell suspension was layered over a Ficoll-Hypaque gradient to yield the cells of interest [15]. T regulatory cells were identified with anti-mouse transcription factor forkhead box P3 (FOXP3) conjugated to allophycocyanin (RnD Systems), and T helper 17 cells were identified with anti-mouse retinoic orphan receptor-gamma (RORγ) conjugated to peridinin-chlorophyll-protein (BD Pharmingen) as previously described [15]. All cells were analyzed using a Beckman Coulter Gallios Flow Cytometer, which acquired 25,000 events per sample.

### Statistical analysis

All data are expressed as a mean ± standard error of the mean when applicable. Two-way ANOVA with Tukey’s post hoc test was used to determine if there was a group (i.e., NP, HELLP, NP+AKI, HELLP+AKI), AKI (i.e., AKI vs. SHAM), or group × AKI effect on the experimental outcomes. Data was analyzed with GraphPad Prism 7.02. Data were considered statistically significant at *p* values < 0.05.

## Results

### IR during pregnancy increases blood pressure, contributes to the development of HELLP syndrome, and decreases pup weight

**Fig. 1:**
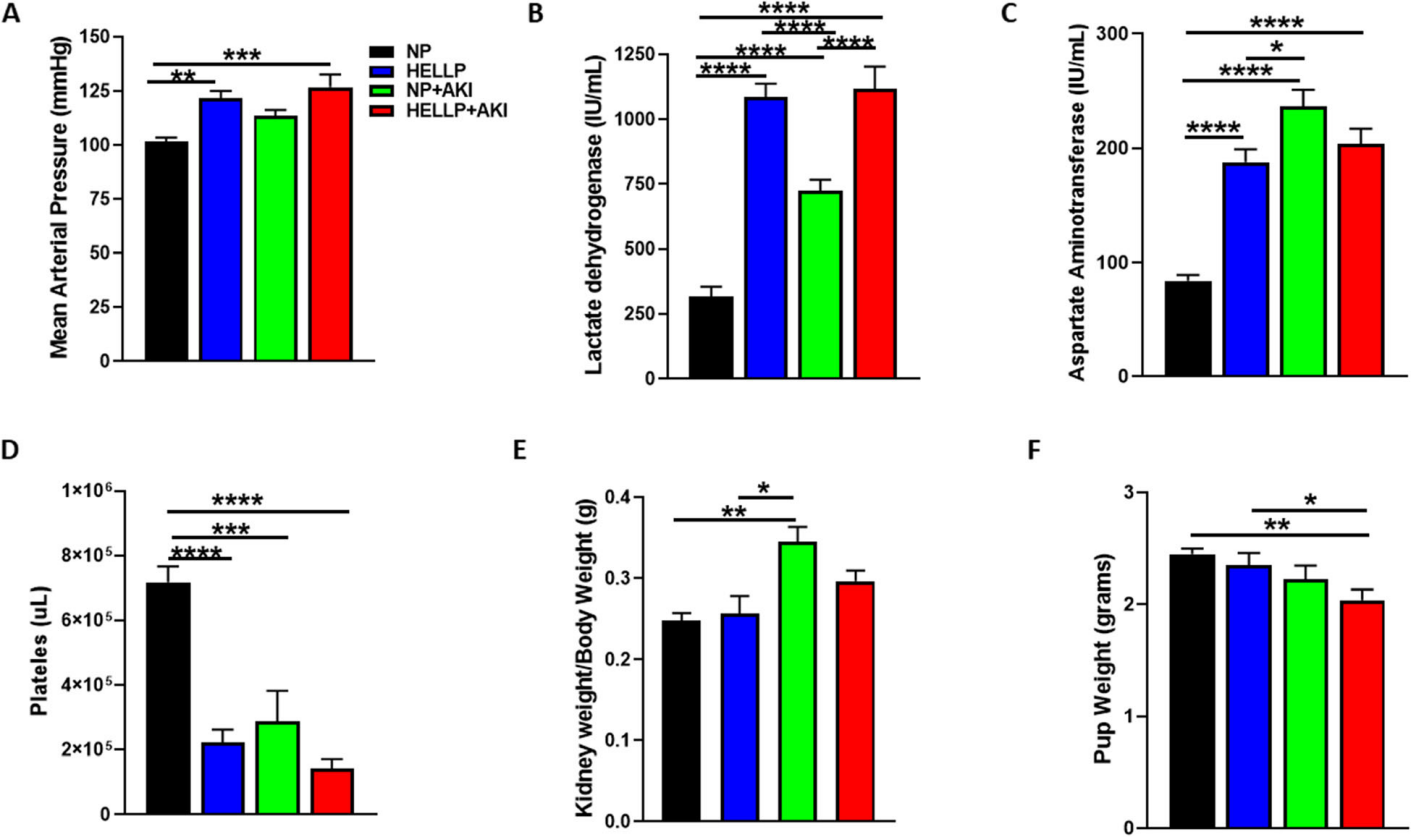
Ischemia reperfusion (IR) during pregnancy increases hypertension and parameters of HELLP in NP rats. Mean arterial pressure was significantly increased in HELLP and HELLP+AKI rats (**a**). HELLP and NP rats subjected to IR had increased lactate dehydrogenase (**b**) and aspartate aminotransferase (**c**) and decreased platelet counts (**d**). The weight of both kidneys per animal was averaged (grams), and the ratio of kidney/body weight (grams) was determined (**e**) as was the average weight of pups per group (**f**). *^–^****p < 0.05–*p* < 0.00005 between indicated groups. *N* = 13–15/group

To determine if IR increases blood pressure during pregnancy, MAP was measured 24 h after AKI induction. There was a significant main group effect on MAP (*F*_1,53_ = 21.06, *p* < 0.0001) which was significantly affected by AKI (*F*_1,53_ = 5.61, *p* = 0.02). Upon post hoc analysis, MAP was significantly increased in HELLP (*p* = 0.001) and HELLP+AKI (*p* = 0.0001) rats compared to NP rats (Fig. 1a). As part of the diagnostic criteria of HELLP syndrome is an increase in hemolysis and liver enzymes and a decrease in platelets, we measured these markers to determine if IR had an effect. There was a significant main group effect on LDH (*F*_1,52_ = 115.8, *p* < 0.0001) which was significantly affected by AKI (*F*_1,52_ = 16.35, *p* = 0.0002). There was also a significant group × AKI effect on LDH levels (*F*_1,52_ = 12.05, *p* = 0.001); with post hoc analysis indicating all groups were significantly increased (*p* < 0.0001) relative to NP rats, additionally HELLP (*p* < 0.0001) and HELLP+AKI (*p* < 0.0001) rats were increased relative to NP+AKI rats (Fig. 1b). There was a significant main group effect on AST (*F*_1,53_ = 8.6, *p* = 0.005) which was significantly affected by AKI (*F*_1,53_ = 48.47, *p* = 0.0001). There was a significant group × AKI effect on AST levels (*F*_1,53_ = 31.83, *p* < 0.0001). Post hoc analysis indicated that all groups were significantly increased relative to NP rats (*p* < 0.0001). Additionally, NP+AKI rats had AST levels significantly higher than HELLP rats (*p* = 0.02; Fig. 1c). Platelets were similarly affected with a significant main group effect (*F*_1,53_ = 26.5, *p* < 0.0001) which was significantly affected by AKI (*F*_1,53_ = 16.77, *p* = 0.0003). There was a significant group × AKI effect (*F*_1,53_ = 7.8, *p* < 0.009). Post hoc analysis indicated that NP rats had significantly increased counts relative to all other groups (*p* < 0.001; Fig. 1d).

To determine if AKI during pregnancy decreased maternal organ weight, the weight of each organ was divided by the rat’s body weight at the time of euthanasia. There were no significant differences between any organ/body ratios except for the kidney, in which there was a significant effect due to AKI (Table 1). Post hoc analysis indicated that the adjusted kidney weights from NP+AKI rats were significantly increased relative to both NP (*p* = 0.002) and HELLP rats (*p* = 0.01; Fig. 1e). When pup weight was evaluated, there was a significant effect of AKI on pup weight, with post hoc analysis indicating that HELLP+AKI pups weighed significantly less at birth compared to both NP (*p* = 0.003) and HELLP rat pups (*p* = 0.03; Fig. 1f).

**Table 1:** The effect of IR during pregnancy with and without HELLP on maternal organs and fetal birthweight and reabsorptions

	DF	Group		AKI		Group × AKI	
		***F***	***p***	***F***	***p***	***F***	***p***
**Maternal body weight**	53	0.26	0.61	2.52	0.12	0.01	0.91
**Body organ/body weight**							
Placenta	53	1.47	0.23	0.52	0.47	2.36	0.13
Kidney	53	.40	0.53	23.42	< 0.001	2.91	0.09
Spleen	53	0.07	0.79	1.04	0.31	1.06	0.31
Liver	53	0.69	0.41	3.06	0.09	0.03	0.87
Brain	53	0.21	0.65	0.02	0.88	0.75	0.39
**Pup weights**							
Pup birthweight	53	3.57	0.06	11.13	0.002	0.40	0.53
No. of fetal reabsorption	53	0.91	0.35	0.94	0.36	0.006	0.94

*DF* degrees of freedom, *F F* value, *p p* value

### IR exacerbates renal injury during both normal pregnancy and HELLP syndrome

**Fig. 2:**
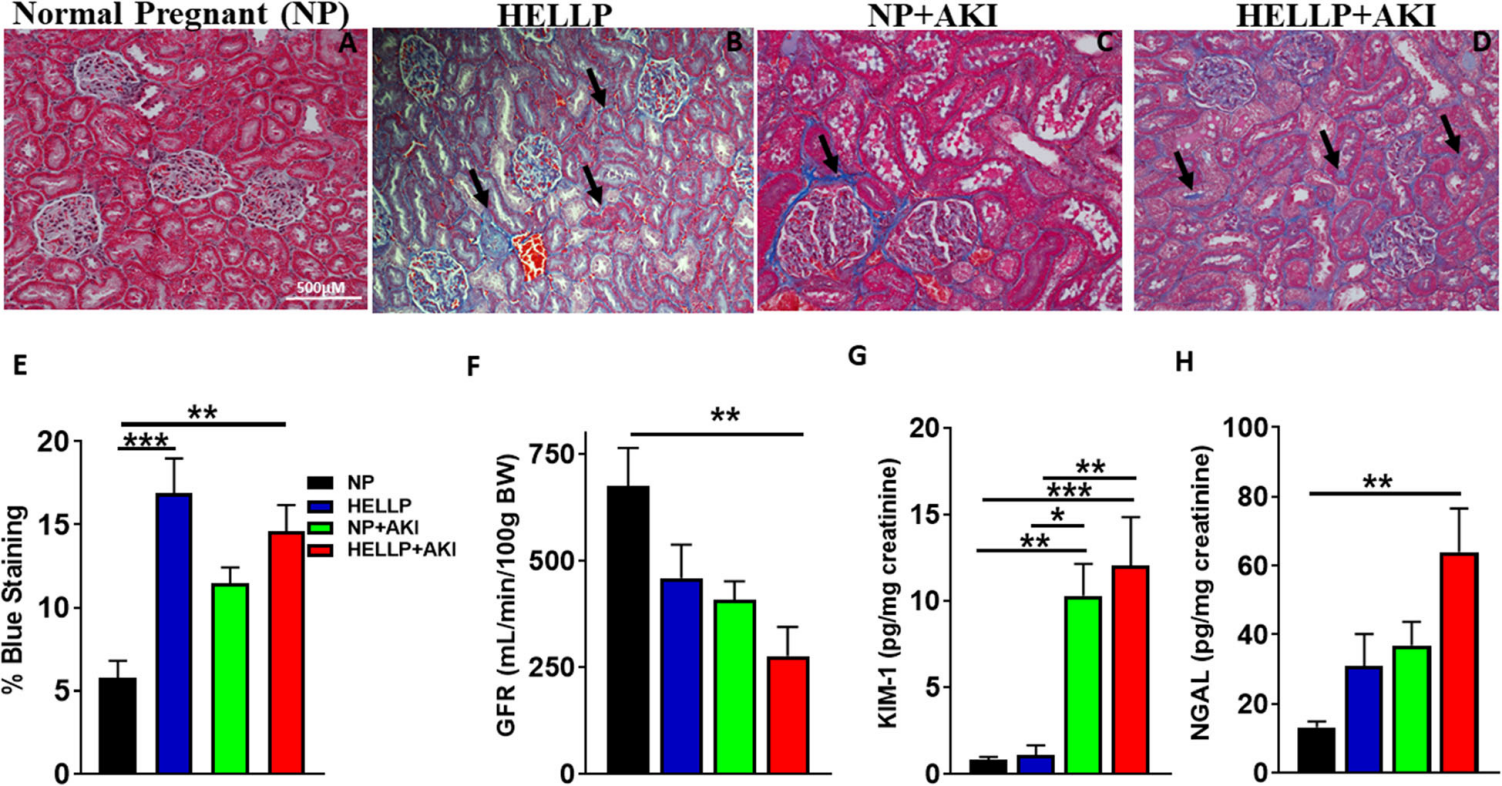
HELLP and ischemia reperfusion (IR) during pregnancy increase renal injury. **a**–**d** Representative pictures from kidneys collected at gestational day 19 (GD19; scale bar is 500 μM). Kidney sections were stained with Mason’s trichrome, and blue areas indicate increased fibrosis (**e**; *n* = 6/group with 10 images per animal scored). A separate group of rats (*n* = 4/group) had renal function assessed by FITC-sinistrin GFR at GD19, and HELLP+AKI rats had significantly less GFR relative to NP rats (**f**). Urinary KIM-1 (**g**) and NGAL (**h**) excretion was increased in response to IR indicative of AKI (*n* = 10/group). Data is expressed as mean ± SEM. *^–^***p < 0.05–*p* < 0.0005 between indicated groups. Black arrows are used to point out areas of increased blue staining

To assess renal injury, renal fibrosis (represented as the percentage of blue staining) was examined in the renal cortex. There was a significant main group effect on renal fibrosis (*F*_1,20_ = 22.99, *p* = 0.0001; representative photomicrographs Fig. 2a–d) and a significant group × AKI effect on renal fibrosis (*F*_1,20_ = 7.06, *p* = 0.02). Post hoc analysis indicated HELLP (*p* = 0.0002) and HELLP+AKI rats (*p* = 0.002) had significantly more renal fibrosis compared to NP rats (Fig. 2e). Renal function was assessed by the clearance of FITC-sinistrin. There was a significant main group effect on GFR (*F*_1,12_ = 5.74, *p* = 0.03) which was significantly affected by AKI (*F*_1,12_ = 9.66, *p* = 0.009). Upon post hoc analysis, GFR was significantly decreased in HELLP+AKI rats compared to NP rats (*p* = 0.01; Fig, 2f). There was a significant AKI effect on KIM-1 excretion (*F*_1,36_ = 28.49, *p* < 0.0001) in which NP+AKI and HELLP+AKI rats excreted significantly more KIM-1 relative to both NP (*p* = 0.003, *p* = 0.0008, respectively) and HELLP rats (*p* = 0.01, *p* = 0.004, respectively; Fig. 2g). There was a significant main group effect on NGAL (*F*_1,36_ = 6.55, *p* = 0.02) which was significantly affected by AKI (*F*_1,36_ = 10.46, *p* = 0.004). Upon post hoc analysis, NGAL excretion was significantly increased in HELLP+AKI rats compared to NP rats (*p* = 0.002; Fig. 2h).

There was a significant main group effect on urine output (*p* = 0.02) which was significantly affected by AKI (*p* = 0.002; Table 2). Post hoc analysis indicated NP rats (*p* < 0.002) had significantly more urine output relative to all other groups. There was a significant AKI effect on albuminuria (*p* = 0.001; Table 2), which post hoc analysis indicated was due to HELLP+AKI rats having significantly more albuminuria relative to NP (*p* = 0.01) and HELLP rats (*p* = 0.02). There was a significant AKI effect on circulating creatinine (*p* < 0.0001; Table 2). Post hoc analysis indicated both NP+AKI and HELLP+AKI rats had significantly increased plasma creatinine relative to NP (*p* = 0.002, *p* = 0.001, respectively) and HELLP rats (*p* = 0.002, *p* = 0.001, respectively). There was a significant main group effect on BUN (*p* = 0.04) which was significantly affected by AKI (*p* = 0.0001; Table 2). Post hoc analysis indicated that NP+AKI rats had significantly higher BUN levels relative to NP (*p* = 0.0004), HELLP (*p* = 0.0003), and HELLP+AKI (*p* = 0.03) rats.

**Table 2:** The effect of IR during pregnancy with and without HELLP on renal parameters

	DF	Group		AKI		Group × AKI	
		***F***	***p***	***F***	***p***	***F***	***p***
Urine output (mL/h)	42	6.34	0.02	10.54	0.002	3.91	0.05
Albuminuria (g/dL)	28	0.98	0.33	13.52	0.001	0.75	0.39
Creatinine (mg/dL)	26	0.10	0.76	35.87	< 0.0001	0.009	0.93
BUN (mg/dL)	27	4.56	0.04	20.09	0.0001	4.16	0.05

*DF* degrees of freedom, *F F* value, *p p* value

### IR during pregnancy is associated with increased infiltrating renal CD4^+^ T cells and decreased T regulatory cells

**Fig. 3:**
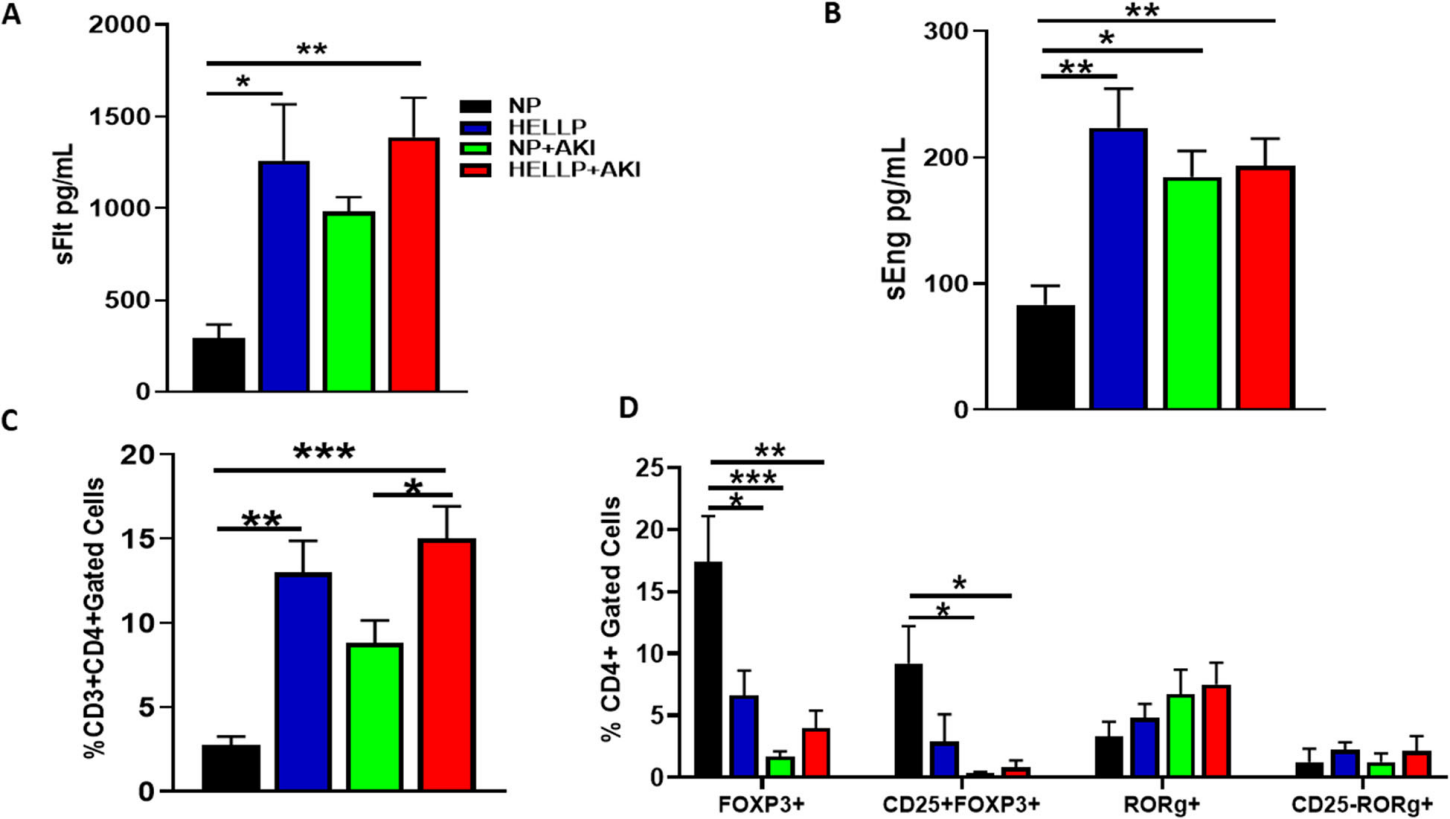
Anti-angiogenic proteins are increased in HELLP and AKI rats. Circulating sFlt-1 (**a**) and sEng (**b**) were significantly increased in HELLP and HELLP+AKI rats compared to NP rats (*n* = 7–8/group). CD3^+^CD4^+^ T cells are increased in HELLP rats and in HELLP+AKI rats between indicated groups (**c**; *n* = 5/group); CD4^+^FOXP3^+^ and T regulatory (CD4^+^CD25^+^FOXP3^+^) cells are significantly increased between the indicated groups whereas there are no differences between in RORγ+ populations (**d**; *n* = 5/group). *^–^***p < 0.05–*p* < 0.0005 between the indicated groups

There was a significant main group effect on circulating levels of sFlt-1 (*F*_1,18_ = 10.40, *p* = 0.05) in which HELLP (*p* = 0.02) and HELLP+AKI (*p* = 0.009) rats had significantly increased sFlt-1 compared to NP rats (Fig. 3a). When circulating sEng was measured, there was a significant main group effect (*F*_1,22_ = 11.22, *p* = 0.003) and a significant group × AKI effect (*F*_1,22_ = 8.74, *p* = 0.007). Upon post hoc analysis, sEng levels were significantly increased in HELLP (*p* = 0.001), NP+AKI (*p* = 0.02), and HELLP+AKI (*p* = 0.007) rats compared to NP rats (Fig. 3b).

There was a significant main group effect on CD3^+^CD4^+^ T cells (*F*_1,16_ = 29.34, *p* < 0.0001) and a significant effect of AKI (*F*_1,16_ = 7.03, *p* = 0.02). Post hoc analysis indicated that this population of cells was significantly increased in HELLP rats (*p* = 0.001) compared to NP rats, and in HELLP+AKI rats compared to both NP (0.0002) and NP+AKI rats (*p* = 0.05; Fig. 3c). Infiltrating T regulatory and T helper 17 cells were also measured in the kidney. There was a significant AKI effect (*F*_1,16_ = 16.77, *p* = 0.0008) and a group × AKI effect on the number of gated CD4^+^FOXP3^+^ T cells (*F*_1,16_ = 8.5, *p* = 0.01). Post hoc analysis indicated that HELLP (*p* = 0.02), NP+AKI (*p* = 0.0007), and HELLP+AKI (*p* = 0.003) rats had significantly decreased cell populations relative to NP rats (Fig. 3d). There was an AKI effect in T regulatory cells (*F*_1,16_ = 8.3, *p* = 0.1), in which both NP+AKI (*p* = 0.02) and HELLP+AKI (*p* = 0.03) rats had significantly decreased T regulatory cells compared to NP rats (Fig. 3d). There were no significant main group or AKI effects on CD4^+^RORγ^+^ cells (*p* = 0.47, *p* = 0.07, respectively) or T helper 17 cells (*p* = 0.34, *p* = 0.97, respectively; Fig. 3d).

## Discussion

There are several animal models of preeclampsia and pregnancy-induced hypertension that demonstrate evidence of renal injury including the reduced uterine perfusion pressure [28, 29], the Dahl S rat [30, 31], the *N*-nitro-l-arginine methyl ester [32], and the transgenic renin-angiotensin model of preeclampsia [33]. There are very few actual models of pregnancy-related AKI. One exception is the model of AKI by Popkov et al. in which pregnant rats underwent unilateral IR on gestational day 18 for 40 min [21]. The study of kidney injury during hypertensive pregnancies such as HELLP syndrome and preeclampsia is important for understanding the mechanisms involved in both the short- and long-term adverse consequences associated with these disorders [5]. The primary goal of the current study was to determine the impact of AKI, on pregnancies complicated with and without HELLP syndrome. We observed that AKI in HELLP rats was associated with decreased GFR and increased urinary levels of KIM-1, NGAL, and albuminuria along with plasma creatinine and increased immune cells, all of which are markers of renal function and/or damage.

During non-pathologic pregnancies, blood volume increases, as does GFR, which contributes to a decrease in serum creatinine and ultimately blood pressure [34]. In the case of HELLP syndrome and preeclampsia, where the kidney is already compromised, it is of no great surprise that these women are at a higher risk of developing AKI compared to their normal pregnant counterparts [1, 10]. In the current study, HELLP rats had a significant increase in renal fibrosis and creatinine and decrease in urine output, indicating a presence of renal injury and a decline in renal function. HELLP+AKI rats had a significant decrease in the same factors as HELLP rats as well as a decrease in GFR and an increase in creatinine, albuminuria, and urinary NGAL and KIM-1.

Blood pressure and the biochemical factors that comprise HELLP syndrome were significantly altered in response to IR in HELLP rats. Interestingly, we observed increases in hemolysis and liver enzymes with a decrease in platelets, which is reminiscent of HELLP syndrome in NP+AKI rats. IR did not increase biochemical parameters of HELLP syndrome in HELLP+AKI rats beyond what was measured in HELLP rats. A recent study by Ye et al. comparing 52 women with HELLP+AKI vs. 56 women with HELLP but not AKI also reported no statistically significant changes in LDH, AST, or platelet levels [9], indicating that AKI in the presence of HELLP syndrome during pregnancy does not worsen these parameters. Low platelets, or thrombocytopenia, are not uncommon among AKI patients, especially those who develop thrombotic microangiopathies during pregnancy [5, 35], but this is to our knowledge the first study indicating that IR conducted during pregnancy can specifically contribute to thrombocytopenia. Pregnancies complicated with thrombocytopenia or thrombotic microangiopathies are also commonly associated with complement activation which has a role in IR, especially in the kidney where this has been proposed to be part of the mechanism leading to the increase in KIM-1 [36]. Complement activation has also been found to be present in pregnancies complicated with AKI and in women with HELLP syndrome, and suppression of this system is one of the few therapies to improve AKI during pregnancy [37, 38].

In our study, there was an increase in sFlt-1 in HELLP and HELLP+AKI rats and an increase in sEng in all rats relative to NP rats. Studies in animal models of sFlt-1-induced preeclampsia have reported kidney damage via glomerular endotheliosis, indicating that infusion of sFlt-1 alone can lead to kidney injury [13, 39]. Likewise, it has been reported that 24 h following IR, male endoglin^+/+^ and endoglin^-/-^ mice have an increase in endoglin expression [18], suggesting that IR can increase endoglin expression. It should also be noted that in a study examining the risk of adverse outcomes among women with preeclampsia or HELLP syndrome, acute renal failure was not associated with increased sEng or an increase in the sFlt-1/PlGF ratio, despite significantly increased levels of sEng and sFlt-1 relative to normal pregnant women [40].

There was a significant number of CD3^+^CD4^+^ T cells in response to HELLP syndrome and in HELLP+AKI rats. This population of immune cells has been proposed to contribute to the progression of AKI to chronic kidney disease (CKD) in both clinical and experimental studies [41, 42]. Despite the significant decrease in T regulatory cells, which are capable of suppressing pro-inflammatory cytokines, there was not a significant effect with AKI or between any of the groups in regard to T helper 17 cells. As HELLP syndrome itself is not consistently associated with decreased or increased T helper 17 cells, this is not surprising [15, 43]; however, more studies are required to determine the role of these immune cells in contributing to the progression to CKD. Renal T regulatory cells have been proposed to decrease CD4^+^ T cell proliferation, decrease subsequent inflammation, and improve/inhibit IR injury through a number of mechanisms [44, 45]. Studies in male mice reported an increase in CD4^+^IL-17^+^ T cells (T helper 17 phenotype) following IR [46, 47]. As T helper 17 cell differentiation is driven in part by transforming growth factor beta [48], it is possible that the increased levels of sEng that are present in HELLP and AKI rats could suppress cellular differentiation. However, that mechanism as well as the presence of IL-17 in this model requires additional investigation.

In this study, we found that NP+AKI rats as well as HELLP+AKI rats also had significant evidence of kidney damage (increased KIM-1, BUN, and creatinine, and decreased urine output) compared to NP rats, which was accompanied by decreased T regulatory cells and decreased sEng. While in the current study we only examined pregnant animal models, it should be mentioned that it has been suggested that the effects of IR on the kidney during pregnancy are less severe compared to what occurs in the non-pregnant state, suggesting that pregnancy increases the tolerance of the kidneys to AKI [21]. Examining AKI in non-pregnant rats is out of the scope of the current study; therefore, we cannot refute this claim. However, we did find that AKI in the presence of existing renal damage, such as that seen in rats with HELLP syndrome, did lead to further exacerbation of injury. This is similar to what has been reported clinically, as clinical studies suggest that women with HELLP syndrome who develop AKI have worse maternal and fetal outcomes compared to women with just HELLP syndrome [10, 12]. It should be noted that it was only rat pups from the HELLP+AKI moms who were significantly underweight at birth when compared to both NP and HELLP litters. There were several limitations to the current study. One of which is the limited number of animals that were successfully used to assess renal function. We also did not examine the placenta to determine if renal IR impaired the placenta or contributed to placental ischemia.

### Perspectives and significance

HELLP syndrome and preeclampsia are severe complications of pregnancy that are also unfortunately associated with an increased risk for the development of AKI [1, 10]. Additionally, what were once thought to be disorders that were resolved shortly after delivery of the placenta, we now know have long-lasting consequences on maternal health [49, 50]. This combined with the increased incidence of pregnancy-related AKI [51, 52] emphasizes the need for adequate animal models to help study the mechanism(s) and potential therapies to further understand the relationship between hypertensive pregnancies and AKI. As IR has been reported to consistently induce acute renal injury in experimental pregnant and non-pregnant animal models, it was used to induce AKI in the current study [21, 22]. We report that IR in the presence of HELLP syndrome exacerbates renal injury, whereas IR in normal pregnant rats (i.e., NP+AKI) increases the biochemical parameters of HELLP syndrome (LDH, AST), decreases platelets, and increases plasma levels of sEng while decreasing renal T regulatory cells.

## Data Availability

The datasets used and/or analyzed during the current study are available from the corresponding author on reasonable request.
